# Metabolomic Prediction of Pregnancy Viability in Superovulated Cattle Embryos and Recipients with Fourier Transform Infrared Spectroscopy

**DOI:** 10.1155/2014/608579

**Published:** 2014-04-15

**Authors:** Marta Muñoz, Asli Uyar, Eva Correia, Claire Ponsart, Catherine Guyader-Joly, Daniel Martínez-Bello, Brigitte Marquant-Le Guienne, Alfonso Fernandez-Gonzalez, Carmen Díez, Jose Nestor Caamaño, Beatriz Trigal, Patrice Humblot, Susana Carrocera, David Martin, Emre Seli, Enrique Gomez

**Affiliations:** ^1^Centro de Biotecnología Animal-SERIDA, Camino de Rioseco 1225, La Olla-Deva, Gijón, 33394 Asturias, Spain; ^2^Department of Obstetrics, Gynecology, and Reproductive Sciences, Yale School of Medicine, 310 Cedar Street, LSOG 304B, New Haven, CT 06520, USA; ^3^Department of Computer Engineering, Okan University, Tuzla, 34959 Istanbul, Turkey; ^4^UNCEIA, Department of Research and Development, 13 rue Jouet, 94704 Maisons Alfort, France; ^5^UNCEIA, Station Expérimentale, 484 Chemin Darefin, 38300 Chateauvillain, France; ^6^U.T.E. Bos-Lugar de Bos, Guísamo, Bergondo, 15640 A Coruña, Spain; ^7^EMBRIOVET S.L., Rois 18 K, Bergondo, 15165 A Coruña, Spain; ^8^Servicios Científico Técnicos (Oviedo), Universidad de Oviedo, Oviedo, 33006 Asturias, Spain; ^9^Department of Clinical Sciences, Swedish University of Agricultural Sciences (SLU), P.O. Box 7054, 75007 Uppsala, Sweden

## Abstract

We analyzed embryo culture medium (CM) and recipient blood plasma using Fourier transform infrared spectroscopy (FTIR) metabolomics to identify spectral models predictive of pregnancy outcome. Embryos collected on Day 6 from superovulated cows in 2 countries were individually cultured in synthetic oviduct fluid medium with BSA for 24 h before embryo transfer. Spent CM, blank controls, and plasma samples (Day 0 and Day 7) were evaluated using FTIR. The spectra obtained were analyzed. The discrimination capability of the classifiers was assessed for accuracy, sensitivity (pregnancy), specificity (nonpregnancy), and area under the ROC curve (AUC). Endpoints considered were Day 60 pregnancy and birth. High AUC was obtained for Day 60 pregnancy in CM within individual laboratories (France AUC = 0.751 ± 0.039, Spain AUC = 0.718 ± 0.024), while cumulative data decreased the AUC (AUC = 0.604  ±  0.029). Predictions for CM at birth were lower than Day 60 pregnancy. Predictions with plasma at birth improved cumulative over individual results (Day 0: France AUC = 0.690 ± 0.044; Spain AUC < 0.55; cumulative AUC = 0.747 ± 0.032). Plasma generally predicted pregnancy and birth better than CM. These first results show that FTIR metabolomics could allow the identification of embryos and recipients with improved pregnancy viability, which may contribute to increasing the efficiency of selection schemes based on ET.

## 1. Introduction

The current increase in the use of embryos selected for their genetic merit and the need for high fertility recipients [1, 2] has made the improvement of pregnancy rates upon embryo transfer (ET) a major objective in cattle farming. Currently, the selection of cow embryos for ET takes place on the basis of morphology and development stage. However, morphological evaluation is a nonobjective method that leads to discrepant judgments among evaluators [3]. Equivocal classification can alter pregnancy rates [4]. Therefore, improving embryo viability prediction would increase the efficiency of ET programs.

Assessment of embryonic quality requests noninvasive or minimally invasive techniques that do not interfere with embryonic development to term. These conditions restrict the number of procedures with practical application for ET programs. In cattle, embryonic cell biopsy is barely used to evaluate incidence of chromosomal abnormality, while it is used to detect and quantify expression of some genes associated with developmental competence [5–7]. However, gene expression techniques are not extensively used on field, and biopsy, when associated with freezing in particular, may compromise embryonic viability [1, 8, 9]. The analysis of the culture medium (CM) that surrounds the embryo represents a noninvasive alternative in the search for markers associated with embryo viability. Single molecules measured in CM in correlation with embryo viability include glucose, lactate and pyruvate [10–12], oxygen [13, 14], amino acids [15, 16], and the proteome profiling [17, 18]. Recently, a noninvasive combined measurement of developmental kinetics and morphology with oxygen consumption allowed a reliable prediction of pregnancy rates from IVP bovine embryos [19].

Together with the particular limitations exposed, the above methods are technically difficult to perform, time-consuming, and expensive or require qualified operators [10–19]. Therefore, more objective and simple approaches are required to accurately predict embryonic viability in cattle.

Classically less attention has been paid to recipient selection in the ET field. In practice, selection of recipients is based on assessment of corpus luteum (CL) function, by rectal palpation or ultrasonography, and progesterone (P4) measurement. Such selection procedures help to increase pregnancy rates but also often exclude too many intrinsically fertile animals [20–24]. Therefore, developing efficient and systematic methods for recipient selection is a challenging and pertinent novel objective for cattle ET technology. Metabolic analysis of serum or plasma provides a global profile of the metabolic status. Blood perfuses essentially all living cells in the body and it carries information on virtually every cell type. Metabolic changes affect nutrient transport from blood to oviductal and uterine fluids [25]. Thus, some compounds of plasma could reflect the ability of a female to act as a high quality embryo-recipient.

A variety of spectral and analytical approaches may allow determination of the metabolites associated with embryo viability and pregnancy outcome [26]. One of the core approaches of metabolomics is metabolic fingerprinting (MF), a spectroscopy profile directly dependent on metabolites present in a sample that can anticipate the likelihood for an event or defined state to occur [27, 28]. In human in vitro fertilization (IVF), after analyzing culture medium by Raman and near-infrared spectroscopy (NIR), the MF obtained predicted viability in oocytes and embryos [29–32]. The results seemed to be not affected by differences in CM, laboratories, and days of embryo development nor by fertilization type (i.e., intracytoplasmic sperm injection versus insemination) [30, 32]. This indicates that IVP embryos bearing a high reproductive potential alter their CM differently compared to embryos that do not lead to pregnancy, although the use of NIR did not lead to increased pregnancy rates in randomized prospective trials [33, 34].

By using another spectroscopy technique, Fourier transform infrared spectroscopy (FTIR), we recently developed a noninvasive system that successfully predicted embryonic sex [35].

In the present study we analyzed plasma by FTIR metabolomics to predict pregnancy viability in recipients of superovulated embryos. In vivo embryo transfer accounts for approximately 65% of the total embryos transferred worldwide (source: IETS Newsletter, Dec. 2013). Therefore improving pregnancy rates by a better selection of embryos and recipients may contribute to increasing significantly the efficiency of selection schemes. The “gold standard” in vivo embryos used were singly cultured in vitro for 24 h and their CM also analyzed, in order to compare the predictive value of recipients and embryos (i.e., plasma versus CM, resp.). On the basis of previous studies with human embryos replicated in different laboratories and culture conditions [30, 32], we carried out our experiments in two laboratories with distinct work procedures.

## 2. Materials and Methods

All experimental procedures were carried out in accordance with the European Community Directive 86/609/EC and were sanctioned by the Animal Research Ethics Committee of SERIDA (licensed 30/01/09).

Animal experiments with in vivo embryo production and ET were performed in independent laboratories from France (UNCEIA, Station Experimentale de Chateauvillain) and Spain (UTE-Bos).

All reagents were purchased from SIGMA (Madrid, Spain) unless otherwise stated.

### 2.1. Production and In Vitro Culture of In Vivo Embryos

In vivo embryo production methods differed between Spanish and French laboratories involved.

In UTE-Bos (Spain), cyclic, Holstein donor cows were synchronized in estrus. Briefly, a progestagen device (PRID; Ceva, France) was inserted in the vagina. On Day (−5), 8 FSH (Folltropin, Bioniche, Canada) decreasing doses were given every 12 h apart. On Days (−3) pm and (−2) am, cows received prostaglandin F_2_ *α* analogue (2 mL Dalmazin; Fatro Ibérica, Spain). On Day (−2) the progestagen device was removed and donors were injected with 2 mL GnRH (Dalmarelin, Fatro Iberica, Spain). On Day 0, animals showing estrus were inseminated twice at 12 h intervals. Inseminations were performed with *n* = 4 bulls.

In UNCEIA (France) cyclic, lactating Holstein donor cows housed in station were synchronized in estrus by using progestagen implants and prostaglandin F_2_ *α* (PG), as prescribed by the manufacturer (Crestar method, Intervet, France). The animals came into heat 48 h after the implant removal (=Day 0). Starting on Day 10, animals were superovulated with a total dose of 500 *μ*g FSH (Stimufol, Reprobiol, Belgium) given as twice daily injections in a decreasing dose 4-day schedule. At the 5th FSH injection, a PG injection was given. Animals showing estrus 48 h after prostaglandin F_2_ *α* injection were inseminated twice at 12 h intervals. Inseminations were performed with *n* = 6 bulls.

In both the UTE-Bos and UNCEIA laboratories embryo recovery was performed by flushing the uterine horns on Day 6 in the cycle. Recovered embryos were in vitro cultured in synthetic oviduct fluid containing amino-acids, citrate, and myo-inositol supplemented with 6 g/L BSA (SOFaaci) in single 12 *μ*L drops for 24 h. Atmosphere conditions were 5% CO_2_ in air (UTE-Bos) and 5% CO_2_, 5% O_2 _in air in UNCEIA. The SOFaaci compounds used were the same in the two laboratories involved, and culture medium in UTE-Bos was prepared in SERIDA. At the end of the culture period (Day 7), embryos were loaded in straws for ET.

### 2.2. Estrus Synchronization of Recipients

In UTE-Bos, heifers were synchronized in estrus with intravaginal progestagen device (PRID ALPHA, Ceva, Barcelona, Spain) for 8 days and a PG analogue (Dalmazin) injected 24 h before progestagen removal. A GnRH analogue (Dalmarelin) was injected at the time of progestagen insertion and on Day 0.

In UNCEIA, heifers were synchronized in estrus with progestagen implant (Crestar, Intervet, France) for 10 days combined with a PG analogue (Estrumate, Intervet, France) injected 24 h before progestagen removal.

### 2.3. Culture Media and Embryo Recovery for Viability Analysis and Embryo Transfer

Spent culture media (10 *μ*L) and blank controls (i.e., droplets incubated without embryos in them; up to 4 blank controls per batch of embryos cultured simultaneously) were collected on Day 7 and stored frozen at −80°C up to FTIR analysis. On Day 7, single embryos were nonsurgically transferred to recipients in the cranial third of the uterine horn ipsilateral to CL under epidural anesthesia.

### 2.4. Recipient Blood Sampling for Plasma Viability Analysis

Blood plasma samples from recipients were taken in EDTA-vacuum tubes from coccygeal vein puncture. Samples were taken at the time of standing estrus (Day 0) and on Day 7 (2–4 h before the ET time). Blood tubes were immediately refrigerated at +4°C, and centrifuged at 2,000 ×g, not later than 30 min after recovery. Supernatant plasma was aliquoted and stored at −80°C up to FTIR analysis.

### 2.5. Pregnancy Diagnosis

In both places and all recipients, pregnancy was diagnosed by transrectal ultrasound scanning on Days 60 ± 2, and birth date registered.

### 2.6. FTIR Metabolomic Analysis

Spent CM and blank samples were analyzed using a Golden-Gate ATR device (diamond crystal) mounted on a Varian 620-IR FTIR spectrophotometer running Varian Resolutions Pro software version 5.0.0.700. 5 *μ*L of the sample was dropped on the ATR diamond and evaporated under a dry N_2_ flow until the FTIR spectrum was stable and different from that of the water. FTIR spectra (16 measurements per sample) were collected in the spectral range between 600 and 4000 cm^−1^, at 5 kHz speed and 4 cm^−1^ resolution. The relative standard deviation was lower than 3% at every wavelength in the range between 600 and 3500 cm^−1^ (excluding the CO_2_ zone).

### 2.7. Spectral Model Development

The FTIR spectra obtained from CM and blood plasma were uploaded to Matlab programming environment (R2011b; The MathWorks, Natick, MA) for data analysis and predictive model development. Two separate datasets were generated considering Day 60 pregnancy and birth endpoints. In each dataset, samples were labeled as 1 and −1 associated with positive and negative outcomes, respectively. Binary (two-class) classification experiments were performed for model development.

The overall study population included spectra obtained from CM of embryos transferred (*n* = 26 in France and *n* = 23 in Spain). Individual spectral profiles were normalized to the control medium to account for possible impact of variations in the culture conditions. Data corresponding to CO_2_ frequency band (2285–2400 cm^−1^) were removed from the analysis. Each sample was then represented as a row vector of spectra data and the corresponding class label. Spectra from Day 0 and Day 7 plasma obtained from recipients transferred were also analyzed.

In a recent study, we performed a benchmarking experiment to assess discrimination capability of a variety of classification algorithms on prediction of embryonic sex using CM spectra (submitted). Among the classifiers tested, k-nearest neighbor (k-NN) provided the highest prediction accuracy. Therefore, we applied k-NN method for viability prediction in this study.

In the distance based local k-NN model the class label of a test sample is decided to be the same as the most frequent class among its *k* neighborhood. k-NN method provides local solutions assuming that samples which are close together in the feature space will belong to the same class. The distances of each test sample to all training samples are calculated and sorted ascendingly. The majority of the class among shortest *k* distances is chosen as the class of the test sample. As the most general distance metric of k-NN algorithm, Euclidean distance was used in the experiments. The Euclidean distance *d*(*p*, *q*) between the two points *p* and *q* in *N* dimensional space is

(1) $$\begin{array}{c} d\left(p,q\right)=sqrt\left(\overset{N}{\underset{i=1}{\sum }}{\left(p_{i}-q_{i}\right)}^{2}\right). \end{array}$$

Specifically, a weighted k-NN approach was applied where the contributions of neighbors to the class choice were weighted by the inverse of distances to the test sample.

The study dataset is a typical example of high dimension low sample size (HDLSS) problem with 49 samples of CM, 96 samples from plasma, and 1704 features, obtained from spectroscopy analysis. We utilized principal component analysis (PCA) to spectra data for dimensionality reduction.

### 2.8. Training and Testing Strategy

We applied 10-fold cross validation training-testing strategy in the classification experiments. The entire dataset was randomly divided into 10 bins. The predictive model was developed on the 9 bins (training samples) and the performance of prediction was assessed on the remaining bin (test samples). In order to overcome sampling bias, the training-testing procedure was repeated 10 times replacing the test samples with a bin from the training samples. The average results obtained from the repeated tests are presented.

The discrimination capability of the classifiers was assessed in terms of accuracy, sensitivity and specificity (i.e., the proportion of correctly detected positive and negative pregnancy outcomes, resp.), and receiver operating characteristics curve (ROC) analysis [36]. The ROC curve plots the* sensitivity* versus* 1-specificity* by adjusting the decision threshold of classification. ROC analysis enables comparison of classifiers using area under the ROC curve (AUC) as the single performance measure where the classifier with the largest AUC dominates the others.

### 2.9. Experimental Design

In Experiment 1, we obtained the metabolomic profile of individually cultured embryos, upon FTIR analysis of frozen/thawed CM samples of 24 h in vitro-cultured, in vivo-derived fresh embryos. Values were normalized versus those of blank samples cultured without embryos. Predictive models compared pregnant versus nonpregnant animals on Day 60 and at birth within all categories of embryos analyzed.

In Experiment 2, we predicted pregnancy success from recipient plasma metabolomic analysis. For normalization purposes, in the absence of blank controls, we included two days of plasma sampling. Our aim was that subtraction of Day 0 and Day 7 plasma values could be an appropriate tool to normalize recipient data between laboratories. Therefore, plasma spectral values were analyzed on Day 0 and Day 7, each being an independent prediction day, and normalized (i.e., Day 7 and Day 0).

### 2.10. Statistics

All spectral model development, data preprocessing steps, and postprediction statistical analysis were performed using Matlab (R2011b; The MathWorks, Natick, MA). Classifier benchmarking tasks were conducted using Weka (Waikato Environment for Knowledge Analysis), an open-source data mining system [37]. The significance of the differences between the predictive spectral models tested was assessed by comparing the associated AUC values using ANOVA or *t*-test when appropriate. A risk alpha of <0.05 was considered significant for the comparisons.

## 3. Results

### 3.1. Embryo Transfer and Pregnancies

Day 6 in vivo recovered early morulae (*n* = 51) were individually cultured for 24 h. Only embryos that developed in vitro up to late morula to expanded blastocyst stages (*n* = 49) were transferred fresh on Day 7 to synchronized recipients in two experimental herds (UNCEIA, *n* = 26, and UTE-Bos, *n* = 23) (Table 3). Samples of CM from all embryos transferred and the corresponding plasma from recipients were recovered and processed. Day 60 pregnancy and birth rates were >50% (Table 3).

### 3.2. Pregnancy Predictions with CM and Recipient Plasma

Day 60 pregnancies in each individual laboratory involved and within cumulative data were higher than predictions at birth (Table 1). However, cumulative predictions at Day 60 from CM were lower than individual laboratory predictions (France: accuracy = 74.6 ± 5.5, AUC = 0.751 ± 0.039; Spain: accuracy: 74.8 ± 3.9, AUC = 0.718 ± 0.024; cumulative: accuracy = 64.4 ± 1.4, AUC = 0.604 ± 0.029).

**Figure 1:**
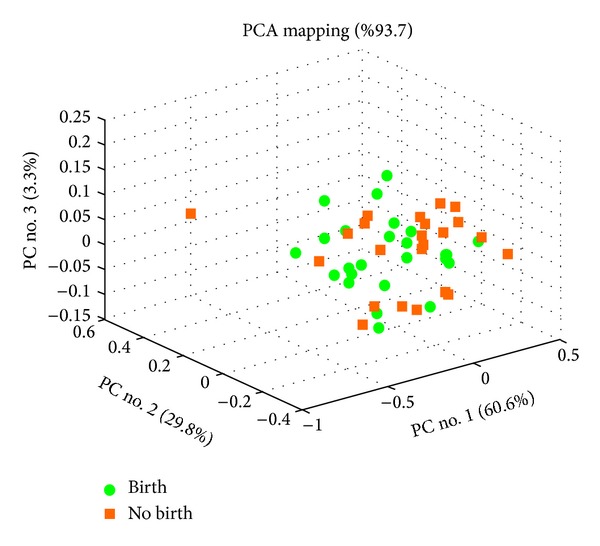
Principal component analysis (PCA) of spectra obtained from Day 0 plasma. The percentages of variability represented by the first three principal components are displayed across PC nos. 1, 2, and 3 on *X*, *Y*, and *Z* axes, respectively.

In contrast, cumulative analysis improved predictions with plasma (Table 2) on Day 60 and at birth when compared to individual results (birth predictions with Day 0 plasma from France: accuracy = 66.4 ± 7.1, AUC = 0.690 ± 0.044; Spain: accuracy and AUC < 0.55; cumulative: accuracy = 72.1 ± 2.0, AUC = 0.747 ± 0.032). Spectra profiles of birth predictions from Day 0 plasma are visualized in a 3D principal component space (Figure 1).

Plasma Day 0 gave AUC and accuracy cumulative values higher than those from Day 0 to Day 7 normalized.

## 4. Discussion

We show here that CM metabolome reflects viability of in vivo embryos and that the metabolic fingerprint of recipient plasma provides robust information on the likelihood of pregnancy and birth. The embryo produced in vitro shows capacity to modify its environment early in development. Thus, in the cow uterus, IVP early embryos trigger detectable maternal responses on Day 8 in the uterine fluid [38], which also changes according to the embryonic sex [39]. Using IVP embryos, sex differences were also captured in the CM with the same FTIR techniques used in this work [35]. In this work, the MF of CM was affected by dissimilar laboratorial procedures, different embryo-donor management, or both. In contrast, results from recipient plasma overcame management differences between the two recipient herds used, leading to accuracy and AUC values, both on Day 60 and at birth (all of them >0.72), higher than those from the two laboratories analyzed separately.

The present findings suggest that the predictive value of CM is limited with in vivo embryos, and future work should consider gaining insight into normalization procedures. Normalization should include classification by embryonic stages with higher sample numbers, and probably culture conditions and time periods other than 24 h.

We did not estimate the effects of 24 h in vitro culture on in vivo embryos after transfer. However, Grade 3 morulae that recovered from superovulated animals yield pregnancy rates similar to noncultured Grade 1 morulae after 24 h in vitro culture [40]. This is consistent with the information from these authors showing that 24 h in vitro culture can be a successful choice for low quality and/or delayed embryos that recovered from flushing.

CM from in vivo embryos was less predictive for birth than for Day 60 pregnancy rates. This is surprising, as recipients carrying in vivo-derived embryos usually show less than 5% embryonic losses from the second month of pregnancy to term [41], a lower rate than IVP embryos [41–43]. Further research is needed so as to investigate whether CM can be representative of these differences between both types of embryos.

The viability profiles of CM with in vivo embryos were not more efficient than conventional selection of embryos. Interestingly, the plasma recipient was generally more predictive of pregnancy success than the CM profile. Within cumulative results, plasma Day 0 gave AUC and accuracy values higher than those from Day 0 to Day 7 normalized, indicating that (combinations of) factors exist in single Day 0 samples able to act as internal controls. Information from recipient (plasma) was generally more predictive of pregnancy success than the embryo CM. Recipient plasma can be affected by different feeding, management, and environmental conditions. However, Day 0 plasma enabled identification of common predictive profiles between recipient herds with an AUC higher than those from each laboratory analyzed apart. These data suggest that two-day sampling could not be necessary, once single plasma samples on Day 0 may provide endogenous normalization.

The ability of a recipient to reach birth has been suggested to be a source of variation higher than the ability of the embryo to survive to term [44–46]. In addition, variation in recipient quality has been shown not to be an important contribution to fetal loss from Day 60 to term [44]. Effective identification of the higher recipient variability by FTIR could explain the superior predictive ability of the animals versus the embryo. It has been predicted that there are intrinsically superior recipients within individual herds [46]. Superior recipient heifers may show changes in endometrial expression of genes and proteins [7, 47–49] affecting major metabolic pathways and immune response. Some ET practitioners are becoming aware of this and retain recipients that successfully delivered calves after ET for future transfers.

To our knowledge, the association of embryos with specific recipients has been not yet studied, and it could help optimize the use of recipient herds. Promising research lines can investigate recipients that are usually discarded for ET using conventional selection criteria. In addition, as recipients and embryos can enter into an early dialogue into which immunological concerns are prominent [38, 39], compatibility between specific embryos (CM) and recipients (plasma) may exist and should be further investigated.

## 5. Concluding Remarks

Selection of in vivo embryos by FTIR analysis of CM to increase pregnancy rates may be performed. Although this approach did not improve the results obtained with conventional selection of in vivo embryos, it is likely that establishing more homogeneous procedures may allow normalization between laboratories leading to improved prediction rates. In contrast, cumulative analysis of recipient plasma from both laboratories identified pregnancy predictive profiles with an AUC higher than those from each laboratory analyzed apart, suggesting that FTIR can be an interesting, simple tool to select recipients on field in conventional MOET programs.

FTIR analysis of CM provides a noninvasive, rapid, and inexpensive method compatible with the highest sanitary standards and international exchanges of embryos. Using embryos and recipients with improved viability indexes will significantly increase pregnancy rates and economic benefit in the cattle breeding industry.

## Figures and Tables

**Table 1:** Day 60 pregnancy and birth predictions on PCA transformed spectra data derived from metabolomic analysis of spent culture medium (CM) of Day 6 in vivo embryos cultured in vitro for 24 h and transferred fresh to recipients on Day 7 in two laboratories (UNCEIA, France, and UTE-Bos, Spain).

Laboratory	Sample analyzed	Pregnancy endpoint	*N*	Positive	Negative	*k*	PCA	Accuracy	Sensitivity	Specificity	AUC
France	Embryo CM	Day 60	26	16	10	3	+	74.6 ± 5.5	75.0 ± 4.2	74.0 ± 8.4	0.751 ± 0.039
		Birth	26	14	12	3	+	68.5 ± 7.6	69.3 ± 7.6	67.5 ± 9.2	0.655 ± 0.075

Spain	Embryo CM	Day 60	23	13	10	3	+	74.8 ± 3.9	67.7 ± 7.1	84.0 ± 6.9	0.718 ± 0.024
		Birth	23	12	11	3	+	66.9 ± 4.2	58.3 ± 6.8	76.3 ± 4.7	0.625 ± 0.032

France + Spain	Embryo CM	Day 60	49	29	20	3	−	64.4 ± 1.4	57.5 ± 2.7	74.4 ± 5.8	0.604 ± 0.029
		Birth	49	26	23	3	−				<0.6

*N*: culture medium or plasma samples analyzed. Positive: samples that correspond to pregnancy/birth. Negative: samples that do not correspond to pregnancy/birth.

*k*: adjustable model parameter of *k*-nearest neighbor classification algorithm.

PCA: principal component analysis (+: applied; −: did not improve the results when applied).

**Table 2:** Day 60 pregnancy and birth predictions on PCA transformed spectra data derived from metabolomic analysis of plasma recovered on Day 0 and Day 7 from recipients prior to transfer on Day 7 of embryos that had been cultured in vitro for 24 h in two laboratories (UNCEIA, France, and UTE-Bos, Spain).

Laboratory	Plasma analyzed	Pregnancy endpoint	*N*	Positive	Negative	*k*	PCA	Accuracy	Sensitivity	Specificity	AUC
France	Day 0	Day 60	25	15	10	3	−	59.6 ± 4.4	58.7 ± 6.9	61.0 ± 5.7	0.657 ± 0.033
		Birth	25	13	12	3	−	66.4 ± 7.1	73.1 ± 15.5	59.1 ± 9.2	0.690 ± 0.044

France	Day 7–Day 0	Day 60	25	15	10	3	−				<0.6
		Birth	25	13	12	3	−	72.0 ± 3.8	69.7 ± 10.2	75.0 ± 8.8	0.789 ± 0.032

Spain	Day 0	Day 60	23	13	10	1	−	69.1 ± 5.2	72.8 ± 9.1	62.0 ± 6.3	0.671 ± 0.067
		Birth	23	12	11	1	−				<0.55

Spain	Day 7–Day 0	Day 60	23	13	10	3	+	68.3 ± 9.6	73.8 ± 15.5	61.0 ± 3.2	0.639 ± 0.047
		Birth	23	12	11	3	+	67.8 ± 3.0	69.2 ± 5.6	66.3 ± 4.4	0.662 ± 0.021

France + Spain	Day 0	Day 60	48	28	20	1	+	74.2 ± 1.1	78.2 ± 3.1	70.7 ± 1.1	0.766 ± 0.014
		Birth	48	25	23	1	+	72.1 ± 2.0	79.2 ± 7.3	64.4 ± 7.0	0.747 ± 0.032

France + Spain	Day 7–Day 0	Day 60	48	28	20	3	+				<0.6
		Birth	48	25	23	3	+	69.8 ± 6.0	58.4 ± 3.4	82.2 ± 9.5	0.657 ± 0.033

*N*: culture medium or plasma samples analyzed. Positive: samples that correspond to pregnancy/birth. Negative: samples that do not correspond to pregnancy/birth.

*k*: adjustable model parameter of *k*-nearest neighbor classification algorithm.

PCA: principal component analysis (+: applied; −: did not improve the results when applied).

Day 7 plasma alone (not represented) yields <0.6 AUC.

**Table 3:** In vitro development and pregnancy rates of Day 6 in vivo embryos that recovered from superovulated, artificially inseminated cows in UNCEIA (France) and UTE-Bos (Spain) followed by a 24 h individual culture step in SOF + 6 g BSA/L prior to embryo transfer.

				Development rates	
Laboratory	*N*1	*N*2	*N*3	Day 60 pregnancy	Calving
France	27	26	26	17 (65%)	16 (61%)
Spain	24	23	23	13 (56%)	12 (52%)

Total	51	49	49	30 (59%)	28 (55%)

*N*1: early embryos, morulae, flushed and cultured in vitro.

*N*2: embryos developed to a transferable stage after a 24 h in vitro culture.

*N*3: embryos transferred to recipients (used once for ET).

France: all embryos were transferred in a single herd (*n* = 5 bulls).

Spain: embryos transferred in 2 herds (*n* = 4 bulls).

## References

[1] Ponsart C, le Bourhis D, Knijn H (2013). Reproductive technologies and genomic selection in dairy cattle. *Reproduction, Fertility and Development*.

[2] Humblot P, le Bourhis D, Fritz S (2010). Reproductive technologies and genomic selection in cattle. *Veterinary Medicine International*.

[3] Farin PW, Britt JH, Shaw DW, Slenning BD (1995). Agreement among evaluators of bovine embryos produced in vivo or in vitro. *Theriogenology*.

[4] Farin PW, Slenning BD, Britt JH (1999). Estimates of pregnancy outcomes based on selection of bovine embryos produced in vivo or in vitro. *Theriogenology*.

[5] El-Sayed A, Hoelker M, Rings F (2006). Large-scale transcriptional analysis of bovine embryo biopsies in relation to pregnancy success after transfer to recipients. *Physiological Genomics*.

[6] Ghanem N, Salilew-Wondim D, Gad A (2011). Bovine blastocysts with developmental competence to term share similar expression of developmentally important genes although derived from different culture environments. *Reproduction*.

[7] Salilew-Wondim D, Hölker M, Rings F (2010). Bovine pretransfer endometrium and embryo transcriptome fingerprints as predictors of pregnancy success after embryo transfer. *Physiological Genomics*.

[8] Alvarez RH, Cardoso CR, Butzke G, Sousa RV (2012). 273 Bovine embryo sexing in field conditions: efficacy of the polymerase chain reaction method and pregnancy rates in dairy herds located in the South and southeast regions of Brazil. *Reproduction, Fertility and Development*.

[9] Mapletoft RJ, Hasler JF (2005). Assisted reproductive technologies in cattle: a review. *OIE Revue Scientifique et Technique*.

[10] Gardner DK, Wale PL, Collins R, Lane M (2011). Glucose consumption of single post-compaction human embryos is predictive of embryo sex and live birth outcome. *Human Reproduction*.

[11] Gardner DK, Lane M, Stevens J, Schoolcraft WB (2001). Noninvasive assessment of human embryo nutrient consumption as a measure of developmental potential. *Fertility and Sterility*.

[12] Urbanski JP, Johnson MT, Craig DD, Potter DL, Gardner DK, Thorsen T (2008). Noninvasive metabolic profiling using microfluidics for analysis of single preimplantation embryos. *Analytical Chemistry*.

[13] Lopes AS, Larsen LH, Ramsing N (2005). Respiration rates of individual bovine in vitro-produced embryos measured with a novel, non-invasive and highly sensitive microsensor system. *Reproduction*.

[14] Lopes AS, Madsen SE, Ramsing NB, Løvendahl P, Greve T, Callesen H (2007). Investigation of respiration of individual bovine embryos produced in vivo and in vitro and correlation with viability following transfer. *Human Reproduction*.

[15] Brison DR, Houghton FD, Falconer D (2004). Identification of viable embryos in IVF by non-invasive measurement of amino acid turnover. *Human Reproduction*.

[16] Sturmey RG, Bermejo-Alvarez P, Gutierrez-Adan A, Rizos D, Leese HJ, Lonergan P (2010). Amino acid metabolism of bovine blastocysts: a biomarker of sex and viability. *Molecular Reproduction and Development*.

[17] Domínguez F, Gadea B, Esteban FJ, Horcajadas JA, Pellicer A, Simón C (2008). Comparative protein-profile analysis of implanted versus non-implanted human blastocysts. *Human Reproduction*.

[18] Katz-Jaffe MG, McReynolds S, Gardner DK, Schoolcraft WB (2009). The role of proteomics in defining the human embryonic secretome. *Molecular Human Reproduction*.

[19] Sugimura S, Akai T, Hashiyada Y (2012). Promising system for selecting healthy in vitro-fertilized embryos in cattle. *PLoS ONE*.

[20] Hidalgo CO, Gómez E, Prieto L (2004). Pregnancy rates and metabolic profiles in cattle treated with propylene glycol prior to embryo transfer. *Theriogenology*.

[21] Humblot P, Perrin J, Jeanguyot N, Nibart M, Thibier M (1987). Effects of age and quality of thawed embryos, synchronization and corpus luteum function on pregnancy rates of bovine embryo recipients. *Theriogenology*.

[22] Siqueira LGB, Torres CAA, Souza ED (2009). Pregnancy rates and corpus luteum-related factors affecting pregnancy establishment in bovine recipients synchronized for fixed-time embryo transfer. *Theriogenology*.

[23] Spell AR, Beal WE, Corah LR, Lamb GC (2001). Evaluating recipient and embryo factors that affect pregnancy rates of embryo transfer in beef cattle. *Theriogenology*.

[24] Yoshida T, Seki M, Watanabe N (2012). Relation of reproductive performances and rectal palpation for luteum function of heifers 7days after estrus. *Animal Science Journal*.

[25] Leese HJ, Hugentobler SA, Gray SM (2008). Female reproductive tract fluids: composition, mechanism of formation and potential role in the developmental origins of health and disease. *Reproduction, Fertility and Development*.

[26] Bromer JG, Seli E (2008). Assessment of embryo viability in assisted reproductive technology: shortcomings of current approaches and the emerging role of metabolomics. *Current Opinion in Obstetrics and Gynecology*.

[27] Ellis DI, Dunn WB, Griffin JL, Allwood JW, Goodacre R (2007). Metabolic fingerprinting as a diagnostic tool. *Pharmacogenomics*.

[28] Ellis DI, Goodacre R (2006). Metabolic fingerprinting in disease diagnosis: biomedical applications of infrared and Raman spectroscopy. *Analyst*.

[29] Nagy ZP, Sakkas D, Behr B (2008). Non-invasive assessment of embryo viability by metabolomic profiling of culture media (‘metabolomics’). *Reproductive BioMedicine Online*.

[30] Scott R, Seli E, Miller K, Sakkas D, Scott K, Burns DH (2008). Noninvasive metabolomic profiling of human embryo culture media using Raman spectroscopy predicts embryonic reproductive potential: a prospective blinded pilot study. *Fertility and Sterility*.

[31] Seli E, Botros L, Sakkas D, Burns DH (2008). Noninvasive metabolomic profiling of embryo culture media using proton nuclear magnetic resonance correlates with reproductive potential of embryos in women undergoing in vitro fertilization. *Fertility and Sterility*.

[32] Seli E, Sakkas D, Scott R, Kwok SC, Rosendahl SM, Burns DH (2007). Noninvasive metabolomic profiling of embryo culture media using Raman and near-infrared spectroscopy correlates with reproductive potential of embryos in women undergoing in vitro fertilization. *Fertility and Sterility*.

[33] Hardarson T, Ahlstrm A, Rogberg L (2012). Non-invasive metabolomic profiling of Day 2 and 5 embryo culture medium: a prospective randomized trial. *Human Reproduction*.

[34] Vergouw CG, Kieslinger DC, Kostelijk EH (2012). Day 3 embryo selection by metabolomic profiling of culture medium with near-infrared spectroscopy as an adjunct to morphology: a randomized controlled trial. *Human Reproduction*.

[35] Muñoz M, Uyar A, Correia E (2014). Non-invasive assessment of embryonic sex in cattle by metabolic fingerprinting of in vitro culture medium. *Metabolomics*.

[36] Uyar A, Seli E (2012). Embryo assessment strategies and their validation for clinical use: a critical analysis of methodology. *Current Opinion in Obstetrics and Gynecology*.

[37] Witten IH, Frank E (2005). *Data Mining: Practical Machine Learning Tools and Techniques*.

[38] Muñoz M, Corrales FJ, Caamaño JN (2012). Proteome of the early embryo-maternal dialogue in the cattle uterus. *Journal of Proteome Research*.

[39] Gómez E, Caamaño JN, Corrales FJ (2013). Embryonic sex induces differential expression of proteins in bovine uterine fluid. *Journal of Proteome Research*.

[40] Alvarez RH, Meneghel M, Martinez AC, Pires RML, Schammass EA (2008). Transfer of bovine blastocysts derived from short-term in vitro culture of low quality morulae produced in vivo. *Reproduction in Domestic Animals*.

[41] Farin PW, Piedrahita JA, Farin CE (2006). Errors in development of fetuses and placentas from in vitro-produced bovine embryos. *Theriogenology*.

[42] Schmidt M, Greve T, Avery B, Beckers JF, Sulon J, Hansen HB (1996). Pregnancies, calves and calf viability after transfer of in vitro produced bovine embryos. *Theriogenology*.

[43] van Wagtendonk-de Leeuw AM, Mullaart E, de Roos APW (2000). Effects of different reproduction techniques: AI, MOET or IVP, on health and welfare of bovine offspring. *Theriogenology*.

[44] McMillan WH (1996). Potential survival rates to term for transferred in vitro and in vivo derived embryos. *Theriogenology*.

[45] McMillan WH (1998). Statistical models predicting embryo survival to term in cattle after embryo transfer. *Theriogenology*.

[46] McMillan WH, Donnison MJ (1999). Understanding maternal contributions to fertility in recipient cattle: development of herds with contrasting pregnancy rates. *Animal Reproduction Science*.

[47] Ledgard AM, Meier S, Peterson AJ (2011). Evaluation of the uterine environment early in pregnancy establishment to characterise cows with a potentially superior ability to support conceptus survival. *Reproduction, Fertility and Development*.

[48] Ponsuksili S, Murani E, Schwerin M, Schellander K, Tesfaye D, Wimmers K (2012). Gene expression and DNA-methylation of bovine pretransfer endometrium depending on its receptivity after in vitro-produced embryo transfer. *PLoS ONE*.

[49] Walker CG, Littlejohn MD, Mitchell MD, Roche JR, Meier S (2012). Endometrial gene expression during early pregnancy differs between fertile and subfertile dairy cow strains. *Physiological Genomics*.

